# Dehydrated husks and cake of prickly pear (*Opuntia ficus-indica*) processing for broiler feed: Effects on growth performance, carcass characteristics, and meat quality

**DOI:** 10.14202/vetworld.2022.551-557

**Published:** 2022-03-09

**Authors:** Imene Cherif, Rafik Arbouche, Yasmine Arbouche, Achour Mennani, Fodil Arbouche

**Affiliations:** 1 Department of Agronomy Science, Faculty of Life and Earth Sciences, University of Chadli Bendjedid, El Tarf 36000 Algeria; 2 Department of Agronomy, Faculty of Life and Earth Sciences, University of Ghardaia, Ghardaïa 47000 Algeria; 3 Department of Agronomy, Faculty of Life Sciences, University of Sétif 1, El Bez, Sétif 19000, Algeria

**Keywords:** broiler feed, prickly pear processing, production cost

## Abstract

**Background and Aim:**

The potential solution is to use agro-industrial by-products as an unconventional source of raw materials for broiler feed. This study aims to determine the effects of substituting prickly pear (FB; *Opuntia ficus-indica*) husks for corn and FB seed cake for soybean meal on the production performance, slaughter characteristics, and chemical composition of broiler meat.

**Materials and Methods:**

Two hundred day-old chicks of equal sex ratio (1:1) of Big Fast strain, weighing on average 37±2g, were randomly divided into four homogeneous groups of 50 subjects each. Each group was subdivided into 10 packs of five animals, which were banded and numbered. Rations with substitution rates of 0%, 10%, 20%, and 30% of corn and soybean meal by dehydrated husks and FB cake were randomly distributed among the groups.

**Results:**

Average daily gains and body weights on 48 days were improved (p<0.05) in 10% and 20% groups, while the 30% group performed identically to the control. Cold carcass yield was optimal in 10% and 20% groups. The liver weight of the experimental groups decreased significantly (p<0.05), while their gizzard weight increased significantly (+24 points). The meat protein rate evolved proportionally to the substitution rate, whereas the fat rate depreciated by up to −1.08 points for the 30% group compared to the control.

**Conclusion:**

Incorporating FB processing by-products into broiler feed at rates of 10% and 20% improves zootechnical performance, carcass yields, and the chemical composition of the meat.

## Introduction

In Algeria, animal production costs depend on imports of raw materials used in food formulas, resulting in meat products influenced by the parity of the Algerian dinar with foreign currencies. Although the factors influencing growth curves [1] and meat quality [2] are dependent on endogenous factors, domestic animal feeding accounts for 70% of the cost price of the products produced [3] and is influenced by world stock exchange prices. The latter results in inflation, making the prices of meat products beyond the reach of the poorest populations. The use of agricultural and agro-industrial by-products in domestic animal rations [4-7] is a method for regulating or even lowering the cost price of these products. The use of by-products from FB processing (*Opuntia ficus-indica* L. mill) is an alternative to consider, considering that this species is well represented in the country’s arid and semi-arid regions (55,671 ha, [8]), is well adapted to a warm environment [9]. Its fruits are used in local culinary customs and the cladodes consumed by the animals living there [10]. Due to its efficiency in converting water into dry matter [11-14], the FB can be considered a versatile crop [15] and a substitute food, thereby ensuring a balance of digestible energy in pet food.

FB is eaten fresh [16], with the pericarp accounting for 33-55%, the pulp for 45-67%, and the seeds for 2-10% [17]. The fruit is also used for producing juice, jellies, jams, vinegar, and oil from the seeds. This agro-industry generates by-products, including husks, seeds, and oilcake, which are a source of environmental pollution because they are not valorized. Kenny [18] estimated that the yield per hectare is between 12 and 30 tons. If we assume that the agro-industry takes care of half of the planted area, that is, 27,836 ha for an average yield of 21 T/ha of fruit, the by-products generated in envelopes (pericarps) (44%) would be 257,205 T, and in seeds (6%) of 35,073 T translatable into oilcake (97%) of 34,021 T. This total quantity of 291,226 T is without context not negligible if it is valorized in animal feed. The studies conducted in Algeria [19] and Egypt [20] used the whole fruit, while Ragab [21] experimented with the fruit envelope with the addition of enzymes.

However, the use of seed cake and dehydrated husks in poultry farming is not studied. Therefore, this study aims to experiment with the by-products of *Opuntia* fruit processing (seed casings and cakes) in the diet of broilers.

## Materials and Methods

### Ethical approval

The present study was conducted after the approval (No. 0035/AEC/2019) of the Institutional Animal Ethics Committee, Agriculture Department of Ghardaia University, Algeria.

### Study period and location

The study was conducted from 14/02/2019 to 10/04/2019. The trial was conducted at a poultry production unit in the municipality of El Kouif Wilaya of Tébessa (Algeria) in a building with a surface area of 80 m², fitted with thermal insulation made of polystyrene panels.

### Animals, diets, and experimental protocol

The study was conducted over a 48-day rearing period in a closed henhouse equipped with pad-cooling fans and humidifiers to ensure good ambient conditions. The litter consisted of sieved wood shavings.

Two hundred day-old chicks of equal sex ratio (1:1) of Big Fast strain, weighing on average 37±2 g, were randomly divided into four homogeneous batches of 50 subjects each. Each batch was subdivided into 10 packs of five animals, which were banded and numbered, forming a control batch and three experimental batches.

The seed cakes and dehydrated FB husks were supplied by an agro-industrial processing unit producing vinegar and FB oil, located in the commune of Sidi Fredj Wilaya of Souk Ahras in Southeast Algeria. The composition of these two by-products was determined in accordance with the Association of Analytical Chemists [22] method (Table-1) [23].

**Table-1:** Chemical composition of prickly pear processing by-products in % DM.

Composition	Seed cake	Dehydrated envelopes
DM	94.3	94.5
Organic matter	98.3	91.3
Crude protein	8.8	5.6
Crude fiber	55	52.3
Fat	2.7	4
Ash	1.7	8.7
Nitrogen-free extract	31.7	29.4
Gross energy (kcal/kg of DM)	4443	4166
Metabolizable energy (kcal/kg of DM)*	1818	1710
Lysine (g/100 g protein)	0.54	0.98
Methionine (g/100 g protein)	0.32	0.52
Cystine (g/100 g protein)	0.31	0.62

* Estimated according to the formula of Carpenter and Clegg[23] with ME (kcal/kg DM)=35.3×P (%)+79.5×F (%)+40.6×NNE (%)+199. ME=Metabolizable energy, CP=Crude protein, F=Fat, NNE=Non-nitrogenous extractive, DM=Dry matter

WUFFDA software [24] was used for broiler feed formulation; four rations were constituted, with 0% (control feed), 20%, 30%, and 40% substitution of soya meal by FB seed cake and corn by their husks for the different rearing phases (Table-2). Rations were distributed randomly to the four broiler groups.

**Table-2:** Formulas (kg/100 kg feed) of starter (1-20 days), grower (21-33 days), and finisher (34-48 days) feed distributed to chickens according to the rate of substitution of corn by prickly pear (FB) husks and soybean meal by FB seed cake.

Composition	Start				Growth				Finishing			
			
Substitution rate (%)	0	10	20	30	0	10	20	30	0	10	20	30
Ingredients												
Corn	61	55	49	43	64	58	51	45	70	63	56	49
Envelopes from FB	0	06	12	18	0	06	13	19	0	07	14	21
Soya cake	30	27	24	21	27	24	22	19	21	19	17	15
FB seed cake	0	03	06	09	0	3	5	08	0	02	4	06
From milling	6	6	6	6	6	6	6	6	6	6	6	6
Bi-calcium phosphorus	1.2	1.2	1.2	1.2	1.2	1.2	1.2	1.2	1.2	1.2	1.2	1.2
CMV	1.25	1.25	1.25	1.25	1	1	1	1	1	1	1	1
L-Lysine	0.15	0.15	0.15	0.15	0.5	0.5	0.5	0.5	0.5	0.5	0.5	0.5
DL-Methionine	0.4	0.4	0.4	0.4	0.3	0.3	0.3	0.3	0.3	0.3	0.3	0.3
Nutrient content in % DM												
Metabolizable energy (kcal/kg of MS)	2890	2877	2847	2837	2840	2822	2812	2800	2990	3093	3120	3192
Crude protein (%)	21	18.2	20.3	20.4	20;3	19.1	19.1	19.1	18	17	17	16.5
Fat content (%)	2.8	5.4	5.2	5.0	2.9	3.2	5.1	4.9	3.0	5.0	4.9	4.7
Ash (%)	3	3.5	3.8	4.09	2.7	3.4	3.7	4.1	2.9	3.2	3.6	4
Crude fiber (%)	3.0	8.7	13.7	17.7	2.9	8.6	13.6	17.5	2.8	8.4	12.8	17.3
Lysine (%)	1.20	9.62	8.66	7.92	1.38	8.82	8.12	7.16	1.22	7.5	6.81	6.11
Methionine (%)	0.72	2.78	2;53	2.27	0.60	2.68	2.44	2.18	0.57	2.38	2.24	2.02

CMV=Cytomegalovirus, DM=Diabetes mellitus

Temperatures ranging between 36 and 38°C were maintained for the first 10 days due to gas powered cattle raisers and a 24 h illumination, which was then decreased to 12 h during the day and 6 h at night. Water and feed were distributed *ad libitum*, and the refusal was weighed daily. Vaccination against Newcastle disease and infectious bronchitis was performed at the 7^th^ and 21^st^ breeding farms and against Gumboro disease after 14 days of age (no booster vaccination). Then, an anticoccidial was administered in drinking water for 2 successive days after 17 and 34 days of age. The animals were individually weighed at on 10, 20, 33, and 48 days of age. Mortality was recorded daily throughout the experiment.

At the end of the rearing period (48 days), 30 chickens selected randomly from each batch were sacrificed. The live weight, the weight of the warm and cold carcass, legs, head, feathers, gizzard, viscera, and liver were weighed. The average daily gain (ADG), average daily intake (ADI), feed, and liver were weighed. The ADG, ADI, and feed conversion ratio were calculated. At 24 h postmortem, the pH was measured by direct insertion (~2 cm deep) of the electrode of a pH meter into the pectoral muscle of quails according to the method of El Rammouz [25]. The ash, protein, and fat content were determined and calculated [22].

### Statistical analysis

Descriptive statistics and single-factor analysis of variance were performed using the Microsoft Excel (Microsoft, USA) sheet. Statistical analysis and comparison of the means between the different diets (control and experimental) were performed by unidirectional analysis of variance using the SPSS software version 21 (IBM Corp., NY, USA). The Student-Newman-Keuls test was applied as a *post hoc* test to estimate the significance or homogeneity between the different subsets (test of comparison between the means). The differences were considered significantwith a 5% risk of error.

## Results

No mortality was recorded during the whole breeding in all groups. During the start-up phase, the 10% group had significantly (p<0.05) greater weights on 10 and 20 days than the other groups, with +24 g and +51 g, respectively, compared to the control group (Table-3). The limit rate of incorporating these two co-products seems to have been reached during this phase and for this parameter.

**Table-3:** Changes in weight growth and ADG (g/d/subject) during the start-up, growth, and finishing phases as a function of substitution rates.

Phase	0	10	20	30	SEM	p-value
Start-up phase						
Initial weight (g)	37.00	37.00	37.00	37.00	
Weight on 10 days (g)	137^b^	161^a^	114^d^	128^c^	2.19	0.001
ADG 1-10 (g/d/subject)	11.1^b^	13.8^a^	8.6^d^	10.1^c^	0.25	0.02
Weight on 20 days (g)	438^b^	489^a^	412^c^	407^c^	9.67	0.01
ADG 11-20 (g/d/subject)	33.4^b^	36.4^a^	33.1^b^	31.0^c^	1.13	0.001
ADG 1-20 (g/d/subject)	21.1^b^	23.8^a^	19.8^c^	19.5^c^	0.36	0.01
Growth phase						
Weight on 33 days (g)	1032^b^	1139^a^	1020^bc^	987^c^	2.21	0.03
ADG 21-33 (g/d/subject)	49.5^b^	54.2^a^	50.6^b^	48.3^b^	1.48	0.01
Finishing phase						
Weight on 48 days (g)	2003^c^	2225^a^	2061^b^	1948^d^	41.43	0.02
ADG 34-48 (g/d/subject)	69.5^c^	77.5^a^	74.3^b^	68.7^c^	1.18	0.02
ADG 1-48 (g/d/subject)	39.7^c^	43.9^a^	41.4^b^	38.7^c^	0.88	0.001

The indices indicate the period in days over which this parameter was calculated. The presence of different letters on the same line indicates a significant difference between diets (p<0.05). ADG=Average daily gain, SEM=Standard error of the mean

For the same batch, the same trend was recorded for the ADG_1-10d_ and ADG_11-20d_ (+2.7 and +3 g/d/subject, respectively) compared to the control. However, the ADG_1-20d_, which reflects the whole start-up phase, showed a significant difference (p<0.05) of +2.7 g/d/subject from the control, but +4.2 g/d/subject on average with the 20% and 30% groups.

During the growth phase, a 10% substitution rate of soybean meal and corn stimulated a significant weight optimization (p<0.05) on 33 days (+107 g compared to the control), generating the maximum ADG_21-33d_ compared to the other groups. The latter were noticeably similar. This leads us to conclude that, although the ADG of 20% and 30% groups is identical to the control during this phase, there is a clear depreciation of the weights on 33 days.

In the finishing phase, the 30% lot achieved a significant minimum value (p<0.05) in live weight and ADG_34-48d_ after 48 days compared to the other groups. The 20% lot produced significantly higher results (p<0.05) than the control lot, while the 10% lot with 2225 g remained significantly dominant (p<0.05), with an optimal ADG_34-48d_ of 77.5 g/d/subject.

The ADG_1-48d_ of 10% and 20% lots showed a higher value than the control lot, with the latter remaining identical to that of the 30% lot. The ADIs of the experimental batches showed less significant results than the control lot during the first 10 days, with a difference of −5 points (Table-4). Between days 11 and 20, the substitution of soybean meal and corn produced a depreciation of the group’s ADI of 20% of −10 g compared to the other groups that showed similar results. It can also be noted that during the 34–48 days interval, 10% and 20% groups produced identical and superior results to the control groups and 30%, with a mean difference of −24 g. All experimental groups were characterized by an influence of the substitution of soybean meal and corn by FB seeds and their husks, especially on the 10% group, giving it optimization of the ADI, for the 30% group, a devaluation of this parameter, and for 20% and control groups, an intermediate and identical value.

**Table-4:** Changes in average daily intake and the feed efficiency as a function of the substitution rate.

Parameter	0	10	20	30	SEM	p-value
Average daily intake (g/subject)						
From 1 to 10 days	270^a^	265^b^	263^b^	263^b^	3.51	0.01
From 11 to 20 days	511^a^	513^a^	504^b^	510^a^	6.82	0.01
From 21 to 33 days	1500	1490	1488	1487	10.24	0.3
From 34 to 48 days	2844^b^	2869^a^	2870^a^	2846^b^	19.56	0.01
From 1 to 48 days	5125^b^	5137^a^	5125^b^	5106^c^	53.8	0.02
Feed efficiency (g/g)						
From 1 to 10 days	2.7^b^	2.1^c^	3.4^a^	2.9^b^	0.253	0.001
From 11 to 20 days	1.7^b^	1.6^b^	1.7^b^	1.9^a^	0.566	0.01
From 21 to 33 days	2.5	2.3	2.4	2.5	0.292	0.38
From 34 to 48 days	2.9^a^	2.6^b^	2.7^b^	2.9^a^	0.127	0.04
From 1 to 48 days	2.6^a^	2.3^b^	2.4^b^	2.6^a^	0.132	0.01

The presence of different letters on the same line indicates a significant difference between the diets (p<0.05). SEM=Standard error of the mean

The feed efficiency of the 10% and 20% groups of substitution of soybean meal by seed cake and cornmeal by FB husks between the 34^th^ and 48^th^ day and from the 1^st^ to the 48^th^ day was significantly similar and the most expressive (p<0.05) compared to the control groups and 30%.

The incorporation of FB seed cake and husks in the broiler diet does not affect the weight of the viscera, head, legs, and feathers (Table-5). The hot carcass weights and yields of 10% and 20% groups had optimal values (p*<*0.05) compared to the control and 30% groups, which had similar values (p<0.05). The cold carcass weights were significantly different (p*<*0.05) between all groups, with the 10% group dominating, resulting in significantly similar (p<0.05) and optimal yields for 10% and 20% groups, with a difference of about +4 points from the control. The 30% group had the minimum value.

**Table-5:** Changes in slaughter parameters and meat chemistry as a function of the substitution rate.

Parameter	0	10	20	30	SEM	p-value
Slaughter parameters						
Live weight (g) (Pv)	2009^c^	2221^a^	2052^b^	1990^c^	119.1	0.01
Hot carcass weight (g)	1406^c^	1597^a^	1487^b^	1398^c^	41.38	0.01
Hot carcass yield %	69.9^b^	72.0^a^	72.5^a^	70.2^b^	0.12	0.01
Cold carcass weight (g)	1265^c^	1494^a^	1391^b^	1222^d^	44.1	0.01
Cold carcass yield%.	63^b^	67.3^a^	67.8^a^	61.4^c^	1.87	0.01
Visceral weight (g)	239	236	235	240	4.1	0.88
Head weight (g)	67	66	68	69	7.84	0.25
Leg weight (g)	100	98	110	100	11.63	0.35
Weight feather (g)	147	146	146	147	11.03	0.81
Weight liver (g) (Pf)	53^a^	47^b^	45^b^	46^b^	7.82	0.03
Pf/Pv ratio (%)	2.6^a^	2.1^b^	2.2^b^	2.3^b^	0.56	0.01
Gizzard weight (g) (Pg)	58^b^	85^a^	82^a^	80^a^	11.06	0.02
Pg/Pv ratio (%)	2.9^b^	3.8^a^	3.9^a^	4.0^a^	0. 87	0.02
Chemical composition of the meat						
pH	6.34^a^	6.31^a^	6.36^a^	6.23^b^	0.034	0.01
Crude protein content	16.36^b^	18.39^a^	18.50^a^	18.77^a^	0.61	0.004
Fat content	3.06^a^	2.87^b^	2.56^c^	1.98^d^	0.183	0.001
Mineral content	0.88^b^	0.98^a^	0.96^a^	0.94^a^	0.148	0.02

The presence of different letters on the same line indicates a significant difference between the diets (p<0.05). SEM=Standard error of the mean

The incorporation of FB seed cake and dehydrated husks resulted in a significant (p<0.05) decrease in liver weight of the experimental groups, with a similar mean value of 46.5 g, resulting in a difference of −6.5 g with respect to the control. The same observation was noted for the Pf/Pv ratios. However, the gizzard weights had an optimal and similar value for the experimental batches (average 82.3 g), resulting in a significant difference (p<0.05) of +24.3 g with respect to the control.

The pH of the meat at 24 h postmortem for the 10% and 20% groups was not influenced by the substitutions, which took on the same value as the control, while the 30% group recorded a minimal result with respect to all the other groups. The experimental groups had significantly similar (p*<*0.05) and maximum protein values, with a significant difference of +2.19 g compared to the control. The mineral content of the experimental groups was significantly influenced (p*<*0.05) by the incorporation of FB seed cake and seed hulls, with an average difference of +0.08 points compared to the control.

## Discussion

The incorporation of the by-product of FB processing (husks) and the by-product of oil extraction from FB seeds (oilcake) did not cause mortality in the experimental groups, as reported by Badr *et al*. [20] in Cobb chickens fed FB husks and Bakr *et al*. [26] in rabbits fed FB zest. However, Moula *et al*. [19] discovered a mortality rate of 10% in broilers fed with 10% FB cladodes in the diet. Ragab [21] discovered a mortality rate of 3.3% in quails fed diets containing 15% FB shells.

Incorporating FB seed husks and seed cakes at a rate of 10% and 20% in broiler chicken feed increased live weight and ADG. This is consistent with the findings of Badr *et al*. [20], who discovered a 5-15% improvement in weight performance due to the substitution of maize by FB envelopes in Cobb broilers. However, Moula *et al*. [19] observed no significant effect on body weight and ADG for a 5% and 10% incorporation of FB cladodes in the broiler feed, in contrast to Ragab [27], who discovered a non-significant effect on body weight on 70 days but a positive effect on ADG in Hy-Line W-36 cocks fed with FB envelopes. Ragab [21] discovered that incorporating 15% and 30% FB envelopes had no effect on body weight and ADG in quails, while Hassan *et al*. [28] reported final weights and ADG with rations containing 25% and 50% FB envelopes in rabbits. These results contradict those reported by El-Neney *et al*. [29], who discovered that a 20% and 30% incorporation of FB envelopes resulted in a significant increase in live weight. In fattening sheep, Islam *et al*. [30] and Anwar *et al*. [31] reported a positive effect on body weight with incorporation rates of 60% and 80%. Throughout the rearing period, the substitution of maize and soybean meal by FB husks and meals, respectively, had a significant effect on ADI and feed conversion of broilers in the 10% and 20% groups. For their part, Badr *et al*. [20] observed in Cobb chickens fed rations containing 5%, 10%, and 15% FB envelopes, ADIs, and consumption indices were lower than in control, as did El-Neney *et al*. [29] for the incorporation of FB envelopes and Zeedan *et al*. [32] for the incorporation of FB cladodes, and in rabbits at levels of 10%, 20%, and 30%.

The incorporation of FB husks and cakes at a rate of 10% and 20% in the feed for broilers increased the weight of hot and cold carcasses, as well as livers and gizzards, but had no significant effect on other slaughter parameters, in contrast to the findings of Ragab [27] in male chicks of line W-36 and Ragab [21] in quails, who discovered no influence of the incorporation of FB envelopes on the slaughter parameters. However, Badr *et al*. [20] observed a significant influence on carcass and offal weights at incorporation rates of 5%, 10%, and 15%. In rabbits, Abu Shammalah [33] discovered that FB envelopes in rabbit rations influence carcass characteristics. The crude protein content of the meat increased while the fat content decreased in proportion to the rates of incorporation of BF envelopes in the rabbit ration, which is consistent with the results reported by El-Neney *et al*. [29] and Zeedan *et al*. [32]. These findings contradict those of Ragab [21,27] and Moula *et al*. [19].

Beyond the partial incorporation thresholds of 10% and 20% corn by husks and soybean meal by FB cakes in broiler feed; these co-products negatively affect weight on 48 days and the GMQ_1-48d_ while increasing the weight of the gizzard and depreciating the quantity ingested. This would be due to the high-fiber content of the two co-products, which would act as an anti-nutritional factor, as reported by Ragab [27], but would not affect the feed efficiency, resulting in higher protein content of the meat due to better digestibility of the two cakes (associative digestibility) as reported by Zaidi *et al*. [34] and Arbouche *et al*. [4].

## Conclusion

Incorporating FB shells and cakes in broiler feed is only feasible at rates of 10% and 20%, which improves zootechnical performance, carcass yield, and the chemical composition of the meat. The incorporation of FB processing by-products into broiler diets is only possible for substitution rates of 10% and 20% of soybean meal by FB meal and of corn by dehydrated FB husks. Future study should be based on to recombine the incorporation of these two byproducts at unequal rates.

## Authors’ Contributions

IC: Prepared the ground conditions and collected the data. YA: Performed the analysis of the data. AM: Carried out the economic analysis. FA: Designed the study and drafted the manuscript. RA: Revised the manuscript. All authors have read and approved the final manuscript.
